# Case Report: Sporadic Burkitt lymphoma misdiagnosed as dental abscess in a 15-year-old girl

**DOI:** 10.12688/f1000research.16390.1

**Published:** 2018-09-28

**Authors:** Marco Cabras, Paolo G. Arduino, Luigi Chiusa, Roberto Broccoletti, Mario Carbone

**Affiliations:** 1 Department of Surgical Sciences, Oral Medicine Section, CIR-Dental School, University of Turin, Turin, 10126, Italy; 2 Pathology Unit, Città della Salute e della Scienza di Torino, Turin, 10126, Italy

**Keywords:** Burkitt lymphoma, dental abscess, oral cavity, paediatric

## Abstract

**Background:** Burkitt lymphoma (BL) is a non-Hodgkin’s B-cell tumor that can be classified into three variants, based on clinical characteristics and epidemiology: endemic, human immunodeficiency-related and sporadic. Oral sporadic BL is quite an unusual entity, with the gastrointestinal trait being often the first site of appearance.

**Clinical finding:** A 15-year-old patient that presented a symptomatic swelling of the right maxilla, unsuccessfully treated as a primary endodontic disease, displaying solid tissue on CT scan, “starry sky” pattern on oral biopsy, multifocal bone and lymph node uptake on PET.

**Diagnoses, interventions, and outcomes:** A diagnosis of stage IV BL was formulated; Rituximab was then administered for three months according to Inter-B-NHL ritux 2010 protocol and CYM (cytarabine and methotrexate) chemotherapy. The patient was followed-up for three years, with no recurrence.

**Conclusion**: It is important for general dental practitioners to suspect a malignancy in the differential diagnosis of unresponsive odontogenic infections in young healthy patients.

## Introduction

Burkitt lymphoma (BL) is a mature, aggressive high-grade B-cell non-Hodgkin’s lymphoma, which occurs in three distinctive subtypes: endemic (African), human immunodeficiency-related and sporadic (nonendemic) ^1^. Endemic BL, which was the first to be described as a “sarcoma” by Denis Burkitt in African children 60 years ago ^2^, occurs mostly among six-year-old males of equatorial Africa and Papua New Guinea, mainly within the maxillofacial complex, with an estimated 50% of cases detected in jaws or facial bones. Sporadic BL is typically observed in Western countries, with a European incidence of 2.2 cases per million, affecting mostly young adult Caucasian males, frequently within the abdomen, particularly in the ileocecal trait ^1^.

The oral cavity is rarely the first site of onset. In this report, we describe the peculiar case of an IV-stage BL arisen as a maxillary swelling in a 15-year-old girl, misdiagnosed at first as endodontic disease.

## Case report

In November 2015, a healthy 15-year-old female was referred to our Department with chief complaint of dull pain on the permanent maxillary right second molar, on whom the general dental practitioner had already performed root canal treatment and administration of two grams of amoxicillin daily for one week, to no avail. Further questioning revealed an associated hypoesthesia of the right lower lip.

**Figure 1.**
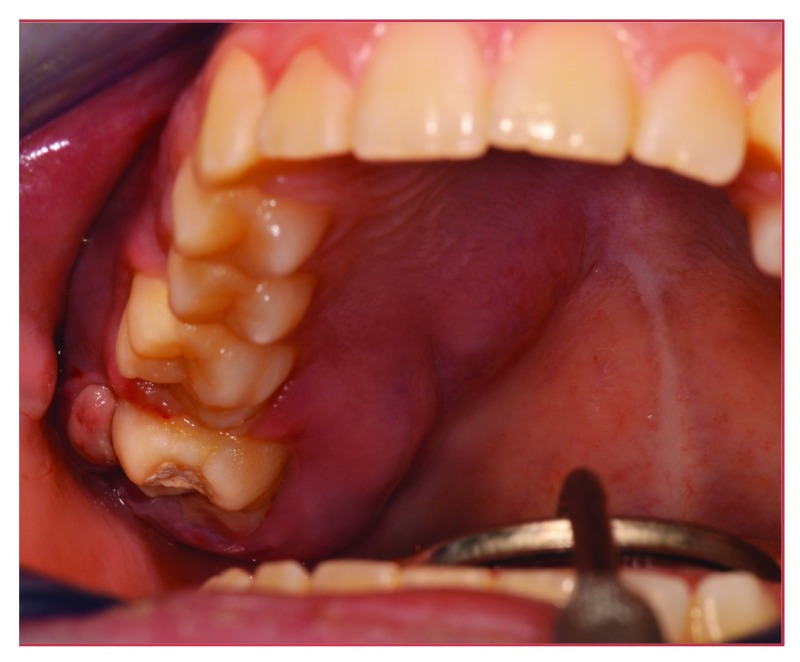
Intra-oral clinical aspect. Swelling of the right maxillary alveolar ridge, expanded to the right hard palate.

**Figure 2.**
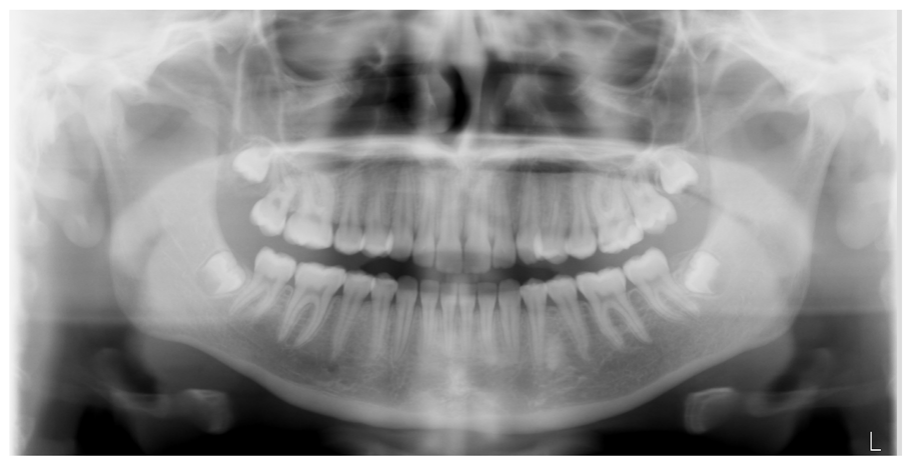
Ortopantomography, showing no evidence of any organic lesion in the oral and maxillofacial area.

Conventional oral examination showed an overall swelling of the gingiva surrounding the painful teeth and extending to the right palatal mucosa ( Figure 1), whereas no signs of oral disease could be detected in the lower lip. Orthopantomography (OPT) showed no signs of odontogenic disease, being completely unremarkable ( Figure 2).

**Figure 3.**
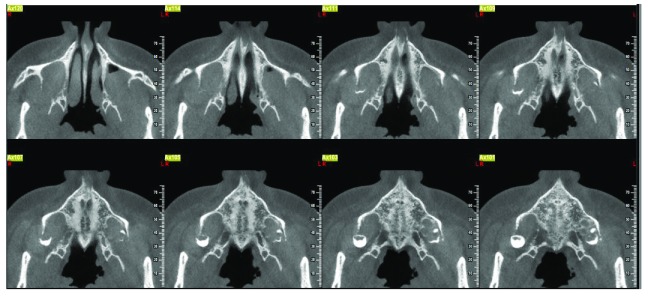
CT scan. Assial view showing solid mass in right maxilla causing destruction of the maxillary sinus floor.

Due to the unreliability of OPT and the unresponsiveness to the combination of root canal and antibiotic treatments, a contrast-enhanced CT scan of the maxillo-facial district was urgently required, revealing solid tissue with high uptake in the right maxilla, with osteolysis and disruption of the floor of the right maxillary sinus ( Figure 3).

**Figure 4.**
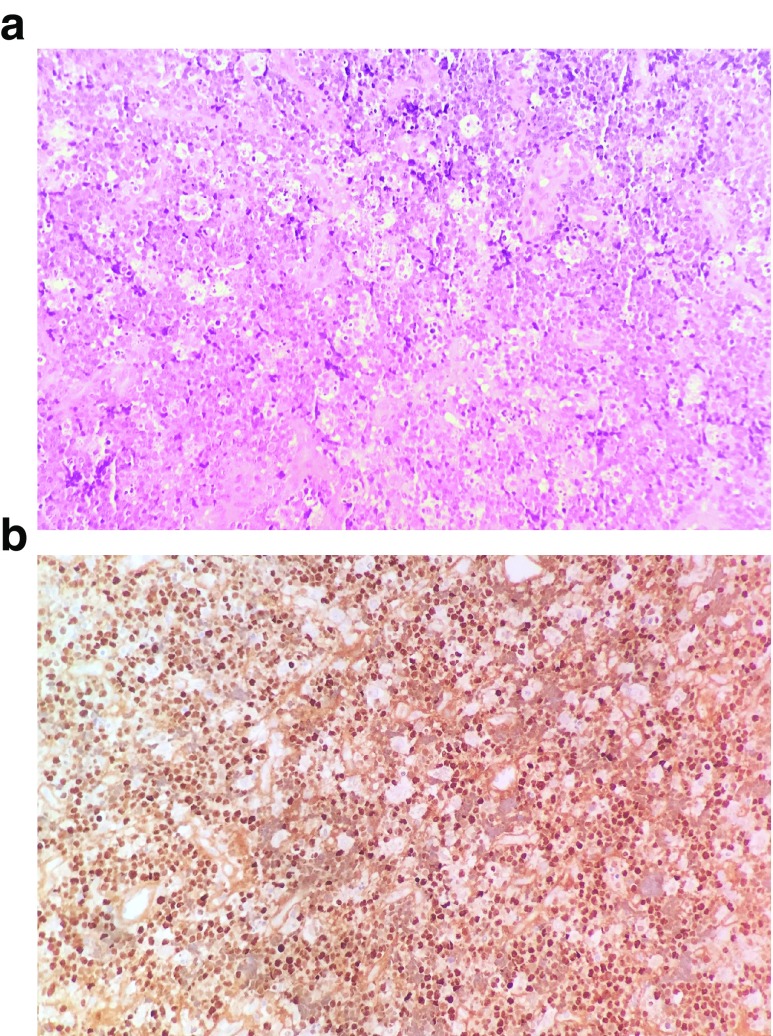
Histopathological examination of the sample. ( **A**) Hematoxylin & eosin 20x revealing “Starry-sky” pattern from pleomorphic and highly apoptotic lymphocytes and macrophages; ( **B**) EBER 20x *in situ* hybridization positive to EBV-encoded RNA.

A field mapping biopsy was conducted, collecting samples from gingiva, inter-radicular tissue from the extracted permanent maxillary right second molar, palatal bone and mucosa. Histology showed in each slide a diffuse infiltration of monomorphic, medium size cells with scarce basophilic cytoplasm, non-cleaved round nuclei, mitotically active with a high rate of spontaneous apoptosis ( Figure 4a). Immunophenotyping revealed an abnormal B-lymphocyte population (75% of cellular events), positive for CD20, with mild co-expression of CD10 c-Myc, and Immunoglobulin lambda light chains. Fluorescent *in situ* hybridization (FISH) revealed IgH/Myc translocation in 70% of the nuclei, being negative for IgH/BCL2 and IgH/BCL6 translocation. CISH staining for EBV-encoded RNA (EBER) transcript was widely positive ( Figure 4b).

The young patient was referred to the Oncohaematology Paediatric Unit of the “Ospedale Infantile Regina Margherita”, Turin, with a clinicopathological diagnosis of Burkitt Lymphoma.

Here, an 18F-FDG PET/CT was required, highlighting intense uptake in maxilla, shoulder blades, right humerus, dorso-lumbar vertebrae and pelvis. Given the combination of clinical signs, microscopy findings and diagnostic imaging, a diagnosis of stage IV Burkitt lymphoma was formulated. The patient was treated with an association of Rituximab according to Inter-B-NHL ritux 2010 protocol and CYM (cytarabine and methotrexate) chemotherapy, between December 2015 and March 2016.

In May 2016, a PET/CT scan showed a complete remission of the disease; clinical oral examination showed the complete remission of gingiva-palatal enlargement. Since then, the currently 18-year-old patient appears to be in good health.

## Discussion

Oral sporadic Burkitt lymphoma (sBL) is a rare clinical entity among children, with few case reports published to date ^3– 13^. To the best of our knowledge, this is the first detailed report of oral sBL in a teenage patient in Italy.

Case-series available worldwide ^14– 18^ show an infrequent involvement of the mouth as the first site, accounting for 3% ^14, 15^, 9.5% ^16^, up to 16% ^17, 18^ of all sBL. Clinically, oral sBL acts as a fast growing, rapidly expanding tumor, which may cause dull, toothache-like pain, teeth malposition, occlusal precontact with subsequent difficulty in chewing and open-bite; ^6^ in some cases sudden teeth loosening can be elicited ^6, 11^.

Such behavior can easily be mistaken for an odontogenic disease, leading to the general dental practitioner (GDP) to administer antibiotics and perform unnecessary procedures, such as root canal treatments of the teeth closest to the swollen or painful area, as the presented case and a previous report have shown ^3^, causal periodontal treatment ^11^ or even teeth extractions ^9, 10^. Moreover, when tonsil is the primary site, dysphagia and obstructive sleep apnoea may occur ^5, 12^.

Panoramic radiograph can be either unremarkable, as in our case and a similar report ^4^, or can reveal an ill-defined isolated ^7, 9^ or multifocal ^11^ radiolucency with loss of lamina dura and periodontal ligament ^6, 7, 9, 13^, sometimes displaying a worrisome “floating teeth” appearance ^6, 7^. Thus, cervicofacial CT is mandatory to properly assess the degree of disruption of the cortical bone and the status of the neighbouring naso-orbital areas ^5, 8, 11^. On the other hand, an 18-FDG PET may be needed either to fulfil the diagnostic work-up when suspecting a widespread lymphoma ^5, 10^, or used as a monitoring tool, especially in the first 12 months after diagnosis, where relapse can be more frequently encountered ^12^. Ultimately, an in-depth histologic analysis, comprehensive of immunophenotyping showing CD20 positive clones and FISH revealing of IgH/Myc translocation will be the key to unravel the differential diagnosis with a wide range of diseases which may share overlapping symptoms and clinico-radiological signs, such as osteomyelitis, benign odontogenic epithelial neoplasms, other types of lymphomas, Langerhans cell histiocytosis, Ewing’s sarcoma, osteosarcoma, chondrosarcoma, neurosarcoma and fibrosarcoma ^4, 7, 10^

Being a rapidly growing tumor with a very high replicative activity, BL proves to be particularly sensitive to cytotoxic chemotherapeutic agents, in particular in a polychemotherapy regimen alongside rituximab, an anti-CD20 monoclonal antibody responsible of a further increase of the five-years survival rate, which can amount up to 90–100% or early stages ^1, 8, 19^.

Therefore, since prognosis is closely related to the earliness of diagnosis, the specialists and GDPs should consider the possibility of a BL in case of rapid swelling of the jaws in young healthy individuals, especially if nonresponsive to traditional antibiotic therapy.

## Consent

An informed consent form was signed by the patient’s mother, in order to obtain permission for photo usage and for the use and publication of the young patient’s data.

## Data availability

All data underlying the results are available as part of the article and no additional source data are required.
