# Supplementary material for: Case Report: Sporadic Burkitt lymphoma misdiagnosed as dental abscess in a 15-year-old girl
Source: F1000Res. 2018 Sep 28;7:1567. [Version 1] doi: 10.12688/f1000research.16390.1 (PMC7029763; doi:10.12688/f1000research.16390.1)
Supplement: Peer review history

# Peer review: Reviewer response for version 1

**DOI:** 10.5256/f1000research.17904.r54889

**Published:** 2020-02-18

**Authors:** Mahmoud Rezk Abdelwahed Hussein

**Affiliations:** 1 Department of Pathology, Assiut University, Assiut, Egypt

This is a well written paper that presents a very interesting case report that concisely describes and addresses a rare clinicopathologic entity in the realm of lymphoma, in particular oral lymphomatous proliferation. The case details were presented clearly and concisely. The relevant literature and studies were nicely summarized and addressed. All in all, I support a decision "to accept".

I confirm that I have read this submission and believe that I have an appropriate level of expertise to confirm that it is of an acceptable scientific standard.

# Peer review: Reviewer response for version 1

**DOI:** 10.5256/f1000research.17904.r40653

**Published:** 2018-12-11

**Authors:** Cristiana Bellan

**Affiliations:** 1 Department of Medical Biotechnologies, University of Siena, Siena, Italy

The Authors describe a case of Sporadic Burkitt lymphoma in an adolescent patient with an "non conventional" oral presentation. They well describe the imaging appearence, the morphology and immunophenotyping as well as molecular characteristics of this entity, highlinting the importance of the differential diagnosis in the oral pathology in young patients.

I confirm that I have read this submission and believe that I have an appropriate level of expertise to confirm that it is of an acceptable scientific standard.
